# From humans to AI: understanding why AI is perceived as the preferred co-creation partner

**DOI:** 10.3389/fpsyg.2025.1695532

**Published:** 2025-12-08

**Authors:** Yuchang Liu, Yongzhong Yang, Haoran Xu

**Affiliations:** 1 School of Public Affairs, Zhejiang Gongshang University, Hangzhou, China; 2 Business School, Sichuan University, Chengdu, China

**Keywords:** artificial intelligence, co-creation intention, perceived novelty, perceived usefulness, the need to belong, creativity theory

## Abstract

With the widespread adoption of generative AI in creative industries, individuals increasingly face a choice between human–human co-creation and human–AI co-creation. Prior comparisons of these modes have largely focused on output quality, efficiency, and user experience, while giving less attention to co-creation intention. Drawing on creativity theory, we argue that perceived novelty and perceived usefulness are the key mechanisms linking co-creator types to co-creation intention, and we test this account across four empirical studies. The results show that, relative to co-creating with humans, co-creating with AI significantly increases participants’ perceived novelty and, counterintuitively, perceived usefulness, thereby increasing co-creation intention. Qualitative interviews identify three principal drivers of why AI is regarded as more useful—efficiency, value, and relationship. Furthermore, we find that the need to belong exerts a moderating effect. Overall, this research extends creativity theory to the AI collaboration context, challenges the conventional assumption that “AI offers greater novelty whereas humans offer greater usefulness,” and uncovers social-motivational boundary conditions in technology-assisted creative work.

## 1 Introduction

The rapid development of generative AI in recent years has triggered a surge of human–AI co-creation (Nah et al., 2023). Co-creation is the process by which different actors interact and collaborate to jointly create content or value (Liu et al., 2024). With the diffusion of tools such as ChatGPT and Midjourney, AI is increasingly becoming an essential partner in creative work—spanning text writing, visual arts, and product design—thereby substantially expanding the boundaries of co-creation (Doshi and Hauser, 2024; Mariani and Dwivedi, 2024; Horton et al., 2023). Against this backdrop, the research scope of co-creation has extended from traditional human–human co-creation to human–AI co-creation, becoming a focal topic for both academia and industry.

Prior work has begun to compare human–human co-creation and human–AI co-creation along multiple dimensions, including creative quality (Tang et al., 2024; Koivisto and Grassini, 2023; Doshi and Hauser, 2024), efficiency (Noy and Zhang, 2023; Koivisto and Grassini, 2023; Anthony et al., 2023), trust mechanisms (Anthony et al., 2023; Kim et al., 2025; Horton et al., 2023), and creative experience and motivation (McGuire et al., 2024; Tang et al., 2024; Wu et al., 2025), and reveals that the two types of co-creation partners are preferred for different outcomes.

Existing research has largely focused on differences at the level of outcomes or process, yet has seldom examined whom individuals actually prefer to partner with when they face a choice among different co-creator types. Preliminary evidence suggests that co-creation preferences are not fixed but are shaped by situational factors and psychological trade-offs (Zhang et al., 2025). However, the lack of systematic inquiry into this issue not only constrains scholarly understanding of the psychological foundations of human–AI co-creation, but also weakens the effective role that AI can play in creative practice. Accordingly, this study centers on a key question: in co-creation tasks, are individuals more inclined to choose a human partner or an AI partner—and what psychological mechanisms underlie this choice?

At its core, co-creation is a creative process, and evaluations of creative quality have long rested on two core dimensions: novelty and usefulness (Runco and Jaeger, 2012). Accordingly, this study adopts creativity theory as its framework and introduces perceived novelty and perceived usefulness as the key mediating mechanisms that explain how different co-creator types shape individuals’ co-creation intention. Equipped with a vast knowledge base and divergent thinking, AI can generate unexpected ideas and thus evoke a stronger sense of novelty (Doshi and Hauser, 2024; Tigre Moura et al., 2023); by contrast, human partners possess distinctive advantages in contextual understanding, commonsense reasoning, and emotional support, thereby enhancing the usefulness and practical value of collaborative outcomes (Huang et al., 2024; Markovitch et al., 2024; Revilla et al., 2023). We therefore infer that different co-creator types influence co-creation intention by altering individuals’ perceived novelty and perceived usefulness.

This research aims to systematically compare human–human co-creation and human–AI co-creation in terms of the mechanisms through which they influence co-creation intention, to identify the mediating roles of perceived novelty and perceived usefulness, and to further examine the moderating role of the need to belong in this process. To this end, we conducted four studies in sequence: Study 1 (*n* = 150) employed a scenario-based experiment to provide an initial test of the effect of co-creator types (human vs. AI) on co-creation intention and to examine mediation via perceived novelty and perceived usefulness. Study 2 (*n* = 243) replicated the findings of Study 1 under a different experimental context and additionally tested self-efficacy as a potential alternative mediator. Study 3 (qualitative interviews, *n* = 50) probed the counterintuitive result from the first two studies—namely, that human–AI co-creation was perceived as higher in perceived usefulness—to uncover its underlying drivers. Study 4 (*n* = 294) built on the preceding findings to further assess whether the need to belong moderates the mediating effect of perceived usefulness.

This study makes three key contributions. First, it integrates humans and AI as two distinct co-creator types within a unified framework, systematically compares their effects on co-creation intention, and uncovers the mediating roles of perceived novelty and perceived usefulness. This not only extends creativity theory to the domain of co-creation but also responds to current calls to elucidate the psychological mechanisms of human–AI co-creation. Second, contrary to the conventional assumption, our results show that AI elevates not only perceived novelty but also perceived usefulness, thereby challenging the entrenched belief that “humans are more useful whereas AI is more novel.” This counterintuitive finding enriches our understanding of AI’s role in creative processes. Third, we verify the moderating effect of the need to belong, revealing the pivotal role of social-motivational factors in technology-assisted creative activities and providing more complete boundary conditions for research on human–AI co-creation.

## 2 Literature review

### 2.1 Human–human co-creation vs. human–AI co-creation

Co-creation typically refers to a process in which two or more actors interact and collaborate to jointly produce content or value (Liu et al., 2024). In conventional settings, co-creation primarily manifests as interpersonal collaboration—namely, human–human co-creation. This mode is widespread in the cultural and creative industries, including design teams (Dubois et al., 2024), advertising creative teams (Klein, 2025), R&D teams (Jeong et al., 2024), and artistic production (Wandel-Brannigan and Matamala, 2024). The advantages of human–human co-creation include pooling diverse perspectives, thereby improving creative quality and originality (Baruah and Green, 2023; Autrey et al., 2024; Grund et al., 2025), and building trust and social capital through sustained interaction, which in turn enhances the quality of collaboration (van Zoonen et al., 2024; Reus et al., 2023) and provides resources for subsequent cooperation and the diffusion of innovation (Pyo et al., 2023; Li et al., 2024).

The rise of generative AI in recent years has broken the constraint of humans as the sole co-creators and opened new perspectives for co-creation research. An expanding body of practice and scholarship now explores human–AI co-creation, such as assisting with copywriting, brainstorming ideas, and drafting design proposals (McGuire et al., 2024; Doshi and Hauser, 2024; Nah et al., 2023). AI can produce diverse sketches and concepts to stimulate designers’ imagination and provide support during evaluation (Yu, 2025), thereby helping to overcome fixation and discover novel solution paths (Yu, 2025; Ma and Huo, 2024). At the same time, AI’s virtually unbounded capacity for content generation markedly improves efficiency (Grewal et al., 2024; Koivisto and Grassini, 2023); for example, in programming tasks, developers can complete work more quickly with AI tools (Wu et al., 2025). These developments have led scholars and practitioners to hold high expectations for human–AI co-creation, viewing it as a promising means to extend the boundaries of human creativity (Wu et al., 2025).

With the accumulation of research, comparisons between human–human co-creation and human–AI co-creation have become a focal topic (see Table 1). Overall, the extant literature does not yield a simple answer to “which mode is superior,” but rather presents a complex and nuanced picture. Human–human co-creation holds advantages in creative quality, affective fulfillment, and trust relationships, tending to generate more original and ingenious ideas (Tang et al., 2024; Koivisto and Grassini, 2023), to spark serendipitous insights through interpersonal interplay and to enhance trust, satisfaction, and creative confidence (Pick et al., 2024; Anthony et al., 2023; Wu et al., 2025), and to strengthen individuals’ sense of ownership and sustained motivation through empathetic feedback (Tang et al., 2024). However, interpersonal collaboration also exhibits limitations—for example, constraints in efficiency and scale and a tendency toward frictions and conformity effects (Longworth et al., 2024; Yu, 2025).

**Table 1:** Human–human co-creation vs. human–AI co-creation.

Comparative dimension	Human–human co-creation	Human–AI co-creation	References
Creative quality	High quality but subjectively variable	Relatively stable quality	Tang et al. (2024), and Doshi and Hauser (2024)
Efficiency	Constrained by the cadence of human thinking and discussion	Able to rapidly generate large volumes of content	Longworth et al. (2024), and Koivisto and Grassini (2023)
Role perception	Viewing the partner as an equal creative agent	Viewing AI as a tool or assistant	Marrone et al. (2024), and Hu et al. (2025)
Communication mode	Relies on shared context and social cues; more flexible and affectively rich	Highly dependent on interface design; intentions must be made explicit	Ma and Huo (2024), Zhou et al. (2023), and Lyu et al. (2022)
Trust mechanism	Grounded in interpersonal relationships and socio-emotional bonds; trust is more stable	Contingent on technical performance; trust is more fragile with a tendency toward low trust	Anthony et al. (2023), Horton et al. (2023), Bellaiche et al. (2023), and Hitsuwari et al. (2023)
Enjoyment	Satisfies social needs and the joy of creating, enhancing enjoyment and engagement	May gradually induce boredom as the task progresses	Rae et al. (2024), Anthony et al. (2023), and Wu et al. (2025)
Creative confidence	Enhances creative confidence	Does not enhance creative confidence	Tang et al. (2024)
Satisfaction	Fosters shared pride/ownership in outcomes, thereby increasing satisfaction	Does not increase satisfaction	Zhang et al. (2024), and Horton et al. (2023)
Creative motivation	Strengthens motivation through emotional resonance and shared accomplishment	Prolonged reliance on AI may reduce a sense of challenge and dampen curiosity	Wu et al. (2025)
Collaborative pressure	Interpersonal frictions may create pressure	Excessive outputs may cause cognitive overload	Yu (2025)
Sense of agency	All members participate in decisions and feel strong ownership of outcomes	Users may see AI as the primary driver and themselves as auxiliary	Tang et al. (2024)

By comparison, human–AI co-creation—enabled by generative AI—exhibits strong efficiency and scalability: it can rapidly produce large volumes of ideas (Koivisto and Grassini, 2023) and, on average, may even be more creative than an individual working alone (Doshi and Hauser, 2024; Wu et al., 2025). Yet its limitations are also salient: its trust foundations are more fragile, hinging primarily on technical performance and thus easily undermined by errors or algorithmic concerns (Horton et al., 2023; Bellaiche et al., 2023; Hitsuwari et al., 2023). Moreover, it lacks the socio-emotional exchange and the sense of agency characteristic of interpersonal collaboration (Ma and Huo, 2024; Zhou et al., 2023; Lyu et al., 2022; Tang et al., 2024).

Given the distinct strengths and limitations of human–human co-creation and human–AI co-creation, a growing research question is: in real-world contexts, whom do people actually prefer to co-create with? Existing studies indicate that such preferences are not fixed but are shaped by specific contexts and psychological trade-offs (Zhang et al., 2025). For example, co-creation intention may be jointly influenced by individuals’ intrinsic motivation (Roy et al., 2023), self-efficacy (Zhang et al., 2025), team climate (Bangun et al., 2023), and the level of trust (Liu et al., 2025). On the one hand, some creative workers—especially younger practitioners in writing and design—exhibit positive attitudes toward human–AI co-creation (Chellappa and Luximon, 2024); on the other hand, many remain cautious or even resistant (Haan, 2023), worrying that AI may undermine creative distinctiveness (Doshi and Hauser, 2024), diminish their sense of accomplishment (McGuire et al., 2024), or threaten job security (Horton et al., 2023). Therefore, whether people prefer to co-create with AI or with humans is not only an open theoretical question but also one of practical importance for optimizing human–AI co-creation and leveraging complementary strengths.

### 2.2 Creativity theory: the mediating roles of perceived novelty and perceived usefulness

Creativity theory holds that novelty and usefulness are the two core dimensions that define creativity (Runco and Jaeger, 2012). In other words, only outcomes that are both novel and useful qualify as truly creative. From this view, perceptions of novelty and usefulness may mediate individuals’ intention to engage in co-creation. When people perceive a co-creation mode as more novel or more useful, they are more likely to initiate and sustain that collaboration (Lenart-Gansiniec et al., 2025; Xie et al., 2023).

#### 2.2.1 The mediating role of perceived novelty

Perceived novelty refers to individuals’ subjective sense of how new an experience or outcome is—namely, the extent to which it feels unprecedented, unfamiliar, and capable of eliciting unexpected stimulation and surprise (Runco and Jaeger, 2012). In the context of co-creation, a high level of perceived novelty means participants regard the focal idea as distinctive and new, thereby becoming more readily engaged and experiencing more positive affect. Compared with human partners, AI—drawing on a vast and heterogeneous knowledge base—can generate nontraditional and even unexpected solutions (Doshi and Hauser, 2024; Tigre Moura et al., 2023); such cross-domain generation often feels refreshing and sparks new inspiration (Chen et al., 2025). For example, Li et al. (2025) shows that customers experience a heightened sense of novelty when robots are involved, which in turn strengthens their willingness to co-create value. Moreover, in human collaboration, individuals often suppress out-of-the-box ideas due to concerns about criticism; when interacting with AI, evaluation apprehension (i.e., the feeling of being judged) is comparatively weaker, making people more willing to propose unconventional ideas and thereby further amplifying perceived novelty (Bullock Muir et al., 2024). Therefore, we infer that, relative to human–human co-creation, human–AI co-creation is more likely to elicit stronger perceived novelty.

Furthermore, perceived novelty is not only a cognitive experience but also a psychological motivator. Motivational theory posits that novelty seeking and exploration are important drivers of intrinsic motivation (Deci and Ryan, 2000; González-Cutre et al., 2016). Prior research shows that perceived novelty can heighten interest and the willingness to persist: for instance, augmented reality (AR) technology increases consumers’ purchase intention by creating unprecedented interactive experiences (Söderström et al., 2024), and in generative-AI applications, novelty is regarded as a key condition for users’ continued use of ChatGPT (Wolf and Maier, 2024). In co-creation settings, novel outcomes elicit curiosity, surprise, and excitement, thereby enhancing positive affect and sustained engagement (Honda and Yanagisawa, 2025). Thus, when co-creation experiences routinely evoke a sense of novelty, participants are more likely to derive enjoyment and a sense of accomplishment and to display higher co-creation intention. Accordingly, we propose:

*H1*: Compared with human–human co-creation, human–AI co-creation increases individuals’ perceived novelty, which in turn enhances co-creation intention.

#### 2.2.2 The mediating role of perceived usefulness

Perceived usefulness typically refers to the extent to which individuals believe that using a particular tool or collaborating with a particular partner will enhance their performance or help them achieve their goals (Davis, 1989). In human–human co-creation settings, human partners hold advantages in contextualized understanding, commonsense reasoning, and the integration of tacit knowledge; they can discern subtle needs, provide immediate feedback, and coordinate the overall direction, thereby minimizing deviations and errors (DeChurch et al., 2024; Sun et al., 2024). This process not only fosters a stronger sense of safety and effectiveness but also heightens participants’ subjective perception of the collaboration’s usefulness. Moreover, human partners can offer emotional support and social feedback during interaction; these non-functional values are likewise foundational to co-creation (Tang et al., 2024; McGuire et al., 2024) and further strengthen participants’ judgments that the collaboration is “worthwhile” (Grenier et al., 2024; Jiang et al., 2024). Therefore, human–human co-creation often yields higher perceived usefulness.

By contrast, although AI enjoys advantages in creativity and efficiency, its recommendations may at times misalign with subtle contextual needs or lack feasibility, necessitating additional screening and verification (Farquhar et al., 2024; Chen et al., 2024). More importantly, behavioral research shows that when people observe algorithmic errors, they tend to exhibit algorithm aversion, becoming more cautious about—and even discounting—subsequent algorithmic suggestions (Kim et al., 2025; Horton et al., 2023). This implies that uncertainty surrounding AI’s reliability and contextual fit may depress individuals’ subjective evaluations of its perceived usefulness.

**Figure 1:**
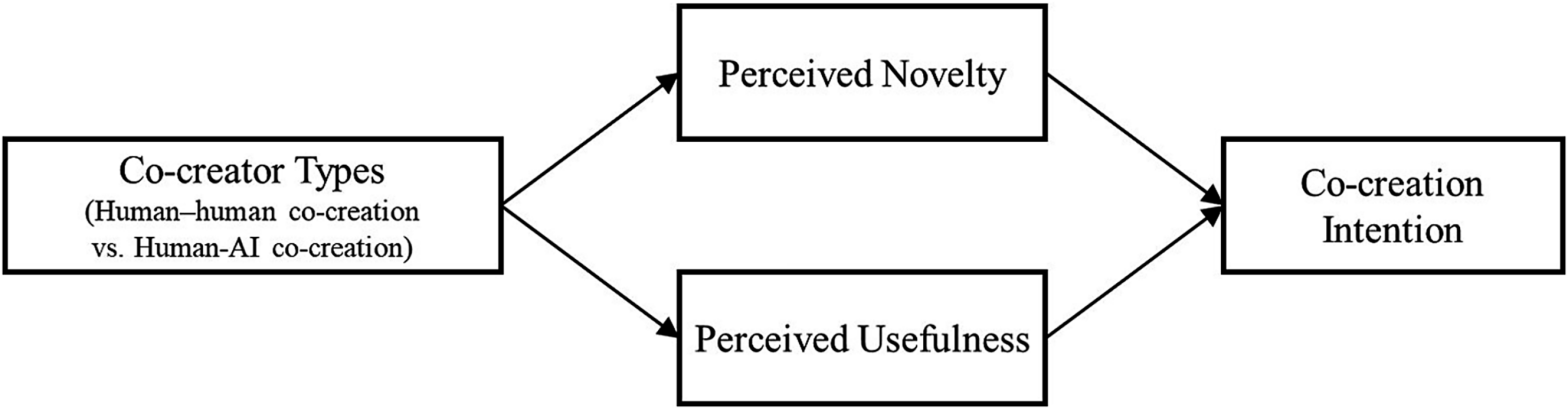
Research model.

From the Technology Acceptance Model (TAM) and its extensions, perceived usefulness has consistently emerged as a key antecedent of adoption and continuance intentions (Davis, 1989; Venkatesh and Davis, 2000; Venkatesh et al., 2003). In co-creation settings, if creative workers believe that a given collaboration mode (e.g., human–human co-creation) can consistently yield more useful outcomes, they are more likely to invest in it and exhibit higher co-creation intention (Liu and Huang, 2025). This logic also accords with the concept of outcome expectations in motivational theory: when people anticipate that collaborating with a particular type of partner will lead to better performance or more useful results, their co-creation intention increases significantly (Bandura, 1991). Accordingly, we propose (see Figure 1):

*H2*: Compared with human–AI co-creation, human–human co-creation increases individuals’ perceived usefulness, which in turn enhances co-creation intention.

## 3 Study 1: effects of co-creator types on co-creation intention

The primary aim of Study 1 is to examine how different co-creator types (human vs. AI) affect individuals’ content co-creation intention, and to test the mediating roles of perceived novelty and perceived usefulness in this relationship.

### 3.1 Experimental design and participants

We employed a single-factor, between-subjects design (co-creator type: human vs. AI). Using G*Power 3.1, the required sample size was estimated at 128 (number of groups = 2, effect size = 0.25, *α* = 0.05, power = 0.80). We recruited 165 participants with prior experience using generative AI via the online platform Credamo.^1^ After attention checks, 150 valid responses remained. Of these, 53.3% were female; 84.6% were aged 21–40; and 81.3% held associate/bachelor’s degrees.

### 3.2 Procedure and measures

Participants were randomly assigned to the human–human co-creation or human–AI co-creation condition and read the corresponding scenario materials. The vignette was self-developed following the recommendations of Lelo de Larrea (2025) and asked participants to imagine engaging in a creative task in which, together with a human partner (vs. AI), they produced a short story including both text and plot. After reading the scenario, participants completed the perceived novelty scale (Yang and Xu, 2025; Cronbach’s *α* = 0.935), the perceived usefulness scale (Davis, 1989; *α* = 0.852), and the co-creation intention scale (You and Robert Jr, 2018; *α* = 0.887). All items were rated on a 7-point Likert scale (1 = strongly disagree, 7 = strongly agree). Finally, participants reported basic demographic information and received CNY 1 as compensation. The full vignette and procedure are provided in Appendix A.

### 3.3 Results

#### 3.3.1 Basic effects tests

**Figure 2:**
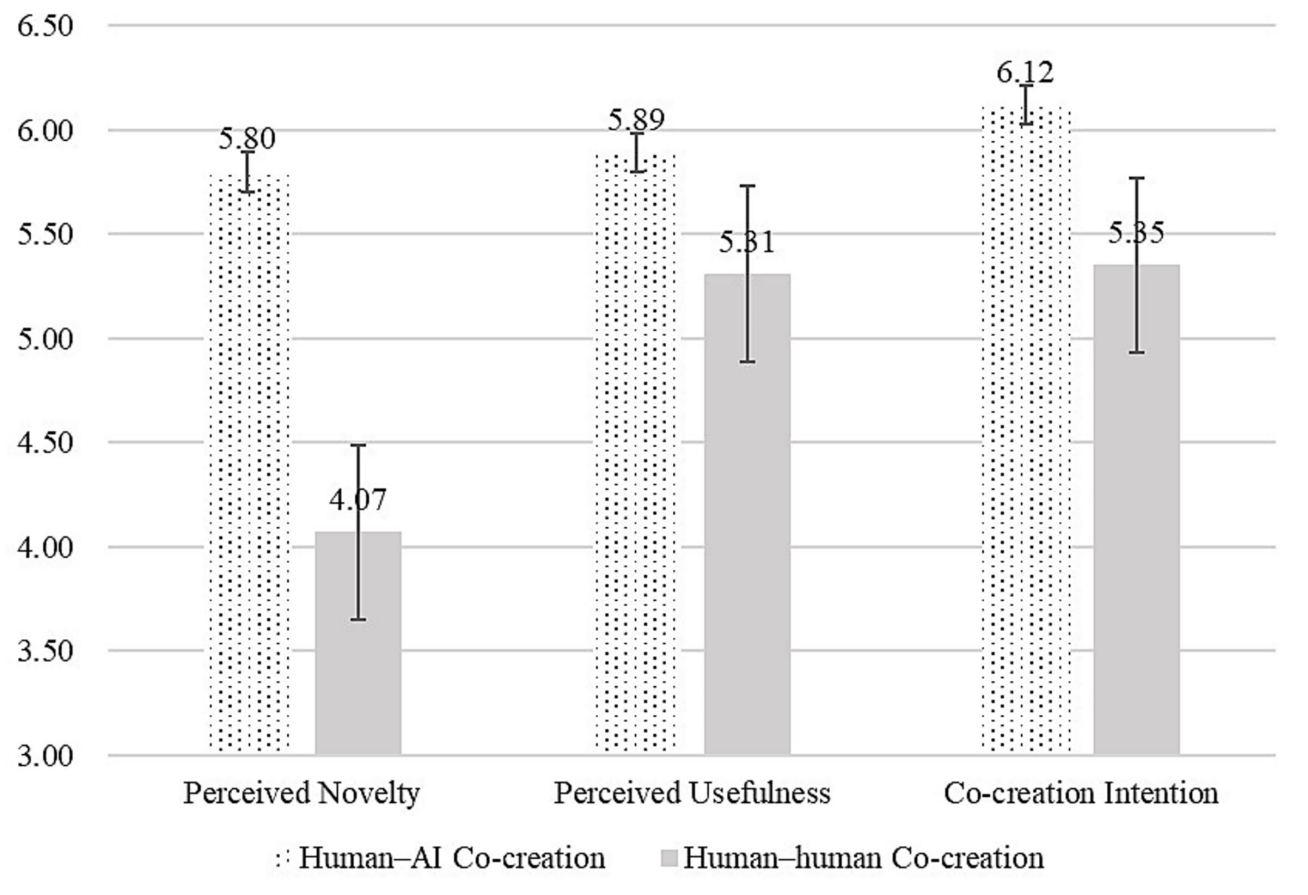
Effects of co-creator types (Study 1).

Using perceived novelty and perceived usefulness as dependent variables, with co-creator type as the independent variable and controlling for gender, age, and education, we conducted ANOVAs. Results showed that, relative to human–human co-creation, human–AI co-creation significantly increased perceived novelty [M_AI = 5.80, SD = 0.86; M_human = 4.07, SD = 1.73; *F*(1,148) = 60.35, *p* < 0.001, *ηp*^2^ = 0.29]. By contrast, human–human co-creation did not increase perceived usefulness relative to AI [M_AI = 5.89, SD = 0.73; M_human = 5.31, SD = 1.20; *F*(1,148) = 13.03, *p* < 0.001, *ηp*^2^ = 0.08]. Human–AI co-creation also significantly increased co-creation intention compared with human–human co-creation [M_AI = 6.12, SD = 0.62; M_human = 5.35, SD = 1.23; *F*(1,148) = 23.29, *p* < 0.001, *ηp*^2^ = 0.14]. Regression analyses with perceived novelty and perceived usefulness predicting co-creation intention indicated that perceived novelty positively predicted co-creation intention [*β* = 0.26, *t*(146) = 5.57, *p* < 0.001], and perceived usefulness also positively predicted co-creation intention [*β* = 0.73, *t*(146) = 17.92, *p* < 0.001] (see Figure 2).

##### 3.3.1.1 Mediation analysis

We used Hayes’s (2017) PROCESS macro (Model 4; bootstrapping = 5,000) to test mediation. The total effect of co-creator type on co-creation intention was significant [*β* = 0.769, SE = 0.159, *t* = 4.83, *p* < 0.001, 95% CI (0.454, 1.084)], whereas the direct effect was not [*β* = 0.041, SE = 0.087, *t* = 0.48, *p* = 0.635, 95% CI (−0.130, 0.212)], indicating full mediation. Further analyses showed a significant indirect effect via perceived novelty [*β* = 0.292, SE = 0.065, 95% CI (0.172, 0.427)] and a significant indirect effect via perceived usefulness [*β* = 0.436, SE = 0.133, 95% CI (0.189, 0.706)]. The difference between the two indirect effects was not significant [*β* = −0.144, SE = 0.133, 95% CI (−0.424, 0.101)]. In sum, Study 1 supports *H1* but not *H2*: relative to human–human co-creation, human–AI co-creation increases both perceived novelty and perceived usefulness, thereby enhancing co-creation intention.

### 3.4 Discussion

The findings indicate that individuals are, overall, more willing to engage in human–AI co-creation. This tendency arises because, relative to human partners, AI more strongly enhances perceived novelty and perceived usefulness, thereby increasing co-creation intention. Notably, the results did not support *H2*; instead, the experiment showed that human–AI co-creation heightened perceived usefulness and significantly promoted co-creation intention. This unexpected pattern reveals a theoretically intriguing phenomenon that warrants further investigation. In addition, prior research suggests that self-efficacy may be an important factor shaping individuals’ willingness to use AI (Zhang et al., 2025; Lin, 2025). Accordingly, Study 2 will formally test the proposed mediation model in a new experimental context and further examine whether task self-efficacy may operate as an alternative mediating mechanism.

## 4 Study 2: replicating the effects of co-creator types on co-creation intention

The purpose of Study 2 is to vary the participant sample and task context to enhance the generalizability of Study 1’s conclusions, and to examine whether task self-efficacy may serve as an alternative mediating explanation.

### 4.1 Experimental design and participants

We employed a single-factor, between-subjects design (co-creator type: human vs. AI). Via the Credamo platform, we recruited 270 participants with prior experience using generative AI; after attention checks, 243 valid responses remained. Among them, 56.8% were female; 86.5% were aged 21–40; and 79.8% held associate/bachelor’s degrees.

### 4.2 Procedure and measures

The procedure closely mirrored Study 1. Participants were randomly assigned to the human–human co-creation or human–AI co-creation condition and read the corresponding scenario. The vignette asked them to imagine undertaking a creative task—designing a promotional poster, including the slogan and layout—together with a human partner (vs. AI) to produce the final work. After reading the scenario, participants completed the perceived novelty scale (Yang and Xu, 2025; Cronbach’s *α* = 0.907), the perceived usefulness scale (Davis, 1989; *α* = 0.717), the co-creation intention scale (You and Robert Jr, 2018; *α* = 0.780), and the task self-efficacy scale (Tierney and Farmer, 2002; *α* = 0.716). Finally, they reported basic demographic information and received CNY 1 as compensation. The full vignette and procedure are provided in Appendix B.

### 4.3 Results

#### 4.3.1 Basic effects tests

**Figure 3:**
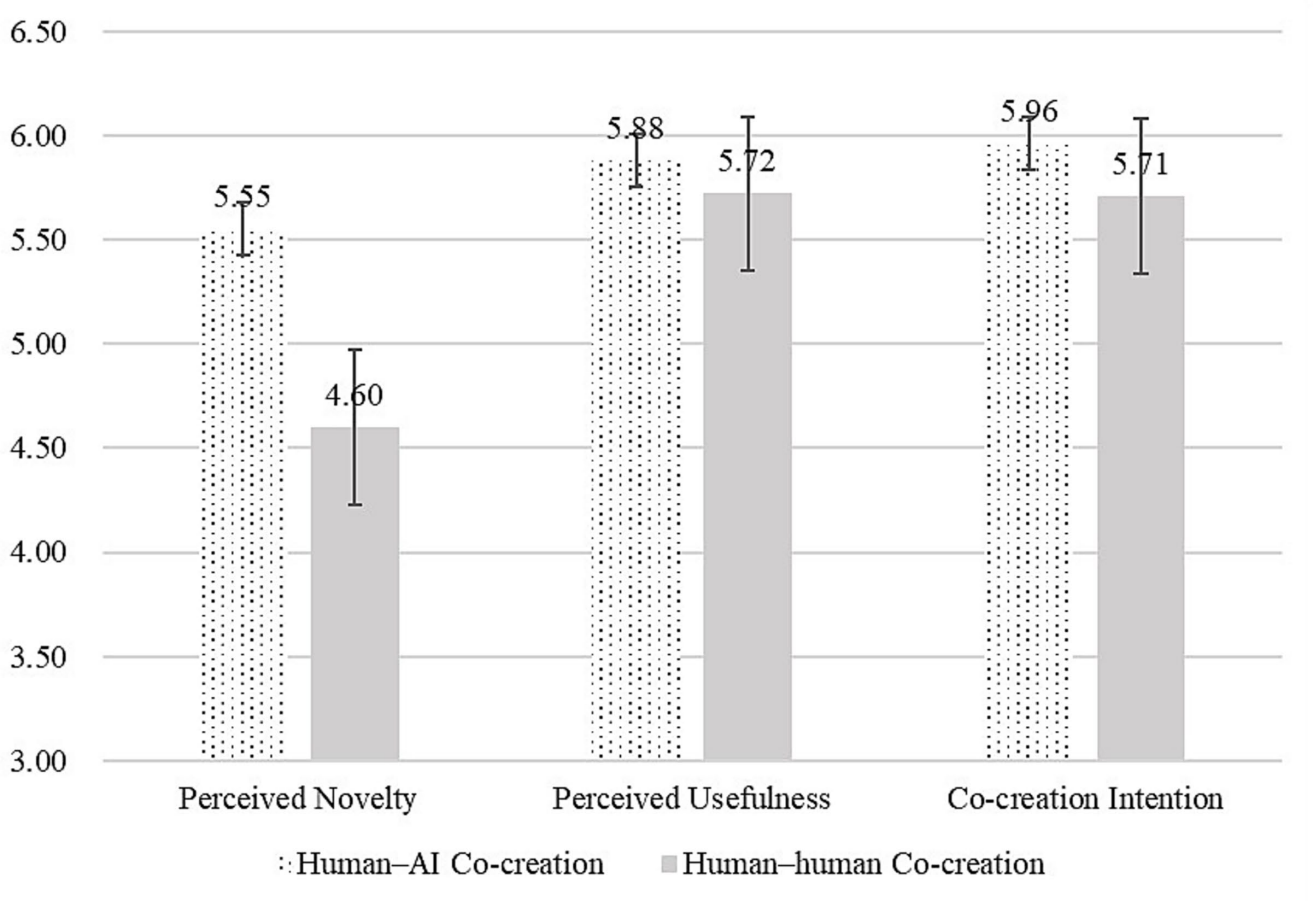
Effects of co-creator types (Study 2).

Using perceived novelty, perceived usefulness, and self-efficacy as dependent variables, with co-creator type as the independent variable and controlling for gender, age, and education, we conducted ANOVAs. Results showed that, relative to human–human co-creation, human–AI co-creation significantly increased perceived novelty [M_AI = 5.55, SD = 0.84; M_human = 4.60, SD = 1.58; *F*(1,241) = 34.92, *p* < 0.001, *ηp*^2^ = 0.13]. By contrast, human–human co-creation did not increase perceived usefulness relative to AI [M_AI = 5.88, SD = 0.60; M_human = 5.72, SD = 0.61; *F*(1,241) = 4.52, *p* = 0.035, *ηp*^2^ = 0.02]. Human–AI co-creation also significantly increased co-creation intention compared with human–human co-creation [M_AI = 5.96, SD = 0.68; M_human = 5.71, SD = 0.77; *F*(1,241) = 7.05, *p* = 0.008, *ηp*^2^ = 0.028]. Regression analyses indicated that perceived novelty positively predicted co-creation intention [*β* = 0.226, *t*(238) = 4.57, *p* < 0.01] and perceived usefulness also positively predicted co-creation intention [*β* = 0.581, *t*(238) = 10.55, *p* < 0.01] (see Figure 3).

##### 4.3.1.1 Mediation analysis

We used Hayes’s (2017) PROCESS macro (Model 4; bootstrapping = 5,000) to test mediation. The total effect of co-creator type on co-creation intention was significant [*β* = 0.248, SE = 0.094, *t* = 2.66, *p* = 0.009, 95% CI (0.064, 0.432)], whereas the direct effect was not [*β* = 0.016, SE = 0.072, *t* = 0.22, *p* = 0.825, 95% CI (−0.125, 0.157)], indicating full mediation. Further analyses showed a significant indirect effect via perceived novelty [*β* = 0.118, SE = 0.031, 95% CI (0.062, 0.182)] and a significant indirect effect via perceived usefulness [*β* = 0.116, SE = 0.058, 95% CI (0.008, 0.230)]; by contrast, the indirect effect via task self-efficacy was not significant [*β* = −0.002, SE = 0.010, 95% CI (−0.024, 0.018)]. Taken together, Study 2 again supported *H1* but not *H2*: consistent with Study 1, human–AI co-creation increased both perceived novelty and perceived usefulness, thereby promoting co-creation intention, while the alternative mediation through task self-efficacy was ruled out.

### 4.4 Discussion

Study 2 replicated the findings of Study 1 in a new task context: *H1* was supported, confirming that human–AI co-creation enhances perceived novelty; however, *H2* was not supported. In contrast, AI co-creation also significantly increased perceived usefulness, further enhancing co-creation intention. Additionally, this study ruled out task self-efficacy as a potential mediator. These results strengthen the robustness of the effects across different creative tasks. Therefore, Study 3 will employ qualitative methods to explore the underlying reasons why people perceive human–AI co-creation as more useful.

## 5 Study 3: exploring why human–AI co-creation is perceived as more useful

Study 3 aims to further validate the findings of Studies 1 and 2 through qualitative research and to explore the key reasons why human–AI co-creation is perceived as more useful.

### 5.1 Research design and participants

This study employed semi-structured online interviews to conduct an exploratory analysis from participants’ subjective experiences. We newly recruited 50 participants, all of whom had firsthand experience co-creating separately with AI and with humans, to enable comparative evaluations of the two forms of co-creation. Among them, 52% were female; 82% were aged 21–40; and 88% held associate/bachelor’s degrees. The interviews used open-ended prompts centered on four core questions: (1) Which do you perceive as more novel—human–AI co-creation or human–human co-creation? (2) Which do you perceive as more useful? (3) With whom are you more willing to co-create? (4) Compared with humans, in what specific ways is human–AI co-creation useful? Please provide at least five descriptors.

### 5.2 Results

Findings from the semi-structured interviews further corroborated the main results of the first two experiments: most participants preferred human–AI co-creation and judged AI superior to human–human co-creation on both perceived novelty and perceived usefulness. Specifically, 76% of respondents (*n* = 38) preferred co-creating with AI, 92% (*n* = 46) viewed AI as more novel, and 74% (*n* = 37) viewed AI as more useful. These results support *H1* and are consistent with the conclusions regarding *H2* in the first two studies.

**Figure 4:**
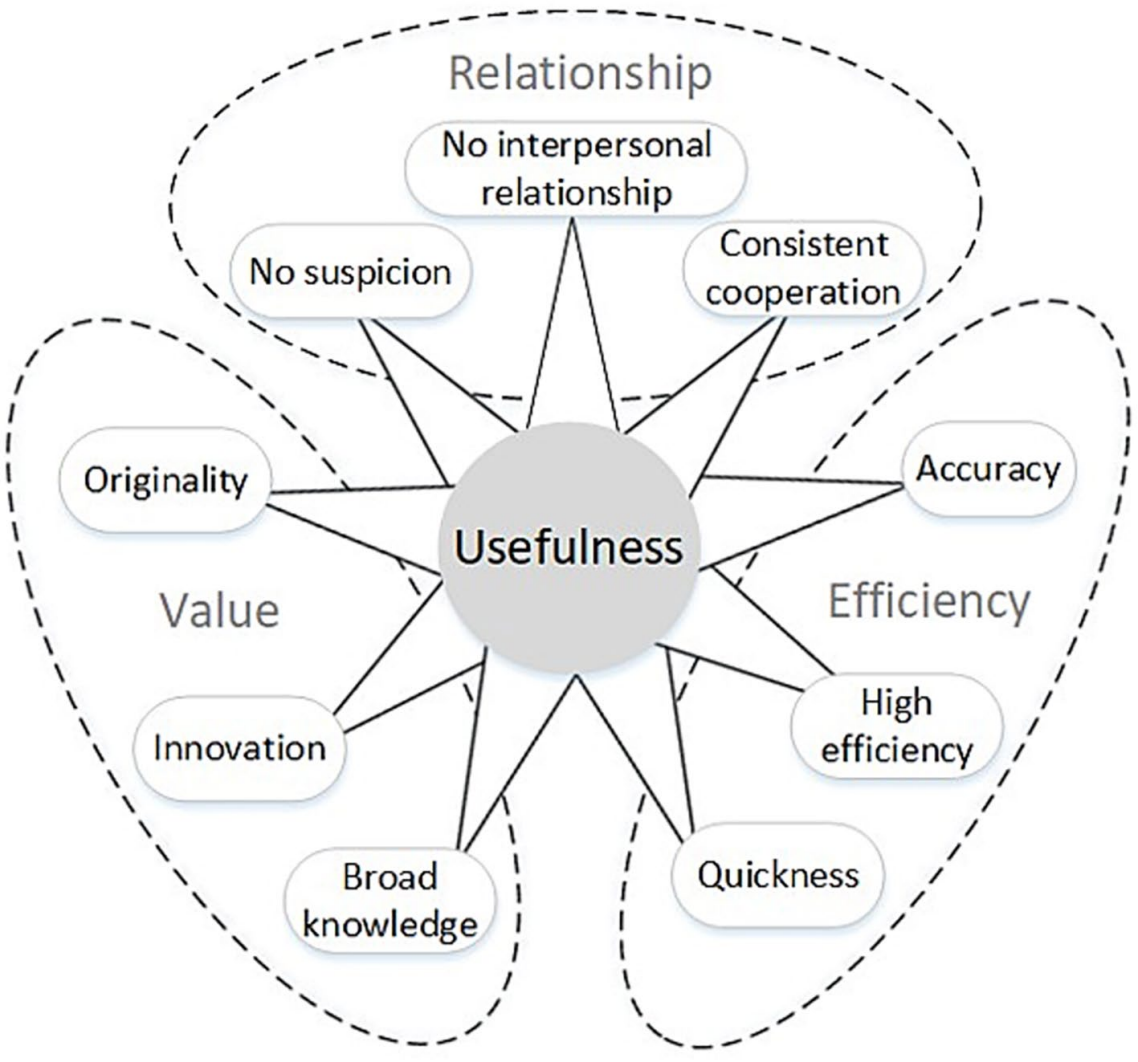
Drivers of AI’s Usefulness.

Drawing on word-frequency analysis and thematic analysis, responses to “Compared with humans, in what ways is human–AI co-creation useful?” clustered strongly around three factors—efficiency, value, and relationship (see Figure 4).

The analyses further revealed three driving factors of AI’s usefulness: efficiency, value, and relationship. Participants widely reported that AI markedly improves work efficiency, with “high efficiency” (*n* = 34), “quickness” (*n* = 18), and “accuracy” (*n* = 15) emerging as the primary usefulness descriptors. In addition, AI’s value for innovation and cross-domain knowledge acquisition was recognized: terms such as “broad knowledge” (*n* = 10), “originality” (*n* = 9), and “innovation” (*n* = 5) reflect AI’s advantages in creative inspiration and breaking mental sets. Finally, AI’s relationship characteristics—captured by “no interpersonal relationship” (*n* = 8), “no suspicion” (*n* = 7), and “consistent cooperation” (*n* = 4)—suggest that collaboration with AI avoids emotional volatility and communication barriers, thereby improving collaborative efficiency.

### 5.3 Discussion

Study 3 focused on clarifying why participants perceive human–AI co-creation as more useful and identified three themes: efficiency, value, and relationship. This aligns closely with current research on the application of AI in creative collaboration.

First, efficiency is among the most important reasons why AI is perceived as highly useful. The study finds that AI can generate a large number of ideas within a very short time, markedly improving work efficiency (Noy and Zhang, 2023). This is consistent with Wu et al. (2025), who show that programmers using AI tools complete tasks significantly faster. In addition, Koivisto and Grassini (2023) note that AI can tirelessly and with great quickness output large volumes of preliminary ideas, substantially enhancing the efficiency and scalability of creative production—a performance advantage that makes AI an ideal partner in the creative process.

Second, value captures AI’s advantages in breaking entrenched mindsets and providing cross-domain knowledge. With a vast knowledge base and diverse modes of thinking, AI furnishes creative workers with perspectives and solution paths that extend beyond everyday experience (Yu, 2025; Ma and Huo, 2024). These attributes—broad knowledge, originality, and innovation—enhance perceived novelty and make AI a pivotal source of creative inspiration. Yu (2025) notes that by generating diverse sketches and concept proposals, AI can stimulate designers’ imagination and offer effective assistance, an inspirational edge that helps overcome fixation and uncover entirely new solutions. Moreover, AI’s value in innovation and knowledge acquisition has been further substantiated in creative and design domains (Ma and Huo, 2024; Mariani and Dwivedi, 2024).

Finally, relationship highlights the distinctive value of human–AI co-creation in “de-interpersonalized” collaboration. Many respondents reported that co-creating with AI avoids the emotional volatility, conflicts of interest, and communication barriers common in human collaboration (Bullock Muir et al., 2024). This mode of no interpersonal relationship allows individuals to focus on the creative task without emotional burdens or interpersonal frictions. Hu et al. (2025) further found that, when collaborating with AI, participants typically regard it as a tool or assistant rather than an equal creative agent, which streamlines the relationship, fosters consistent cooperation, and helps improve task completion efficiency. Koivisto and Grassini (2023) likewise noted that AI can circumvent affective interference that may arise in human collaboration, making the cooperative process smoother.

## 6 Study 4: the moderating role of the need to belong

Although AI has demonstrated significant advantages in many areas, human collaboration still holds unique value in terms of emotional support, creative motivation, and shared sense of accomplishment (Tang et al., 2024; McGuire et al., 2024). However, as Wu et al. (2025) pointed out, prolonged reliance on AI may reduce individuals’ sense of challenge and weaken curiosity and creative motivation. Additionally, Tang et al. (2024) found that participants collaborating with AI did not experience significant increases in creative confidence or creative motivation, in stark contrast to those collaborating with humans. This disparity suggests that individuals’ social needs may play an important role in influencing their preferences and engagement in collaborative efforts. Specifically, in creative collaboration, the emotional bonds and social interactions between humans often serve to spark higher levels of creative motivation and shared sense of achievement.

A growing body of empirical research on co-creation has incorporated social motivational factors, as briefly summarized in Table 2. In human–human co-creation research, the need to belong has frequently been recognized as a key factor influencing co-creative behavior. The need to belong is an intrinsic social motive where individuals desire to form close interpersonal relationships (Baumeister and Leary, 1995), and it significantly influences people’s preferences and engagement in collaborative efforts (Afota et al., 2024). However, in studies on human–AI co-creation or comparisons between two co-creation types, while many have examined social motivational factors such as trust (Daly et al., 2025), empathy (Zhu et al., 2025; Qian and Wan, 2025), reliance (Siemon et al., 2025) and commitment feeling (Wang et al., 2025), the need to belong, although a fundamental social motivation, has rarely been directly investigated or has been treated only as a mediating variable. Building on these observations, this study introduces the need to belong as a moderator to help uncover individual differences in response to different co-creation types and to further clarify the boundary conditions under which social motivations operate in human–AI co-creation.

**Table 2:** Empirical studies on social motivation in co-creation.

Co-creation type	Method	Key social motivational factors	Main findings	Limitations	References
Human–AI co-creation	Survey	Trust	Trust facilitates human–AI collaboration, enhances potential and the AI willingness.	The research focused on trust, neglecting social motivations like affiliation needs and peer belongingness.	Daly et al. (2025)
Human–AI co-creation	Vignette experiment	Artificial empathy	Robots empathy increase emotional connection and satisfaction	Without considering more motivation factors.	Zhu et al. (2025)
Human–AI co-creation	Vignette experiment	Face-related motivation, commitment feeling	Face-giving service robots enhance co-creation intention, mediated by commitment feeling.	Focused on politeness strategies and co-creation, but did not examine more factors.	Wang et al. (2025)
Human–AI co-creation	Vignette experiment	Social bonding and empathy	Co-experiencing with AI enhances the sense of social connection and empathy towards AI.	Not examine belongingness and connectedness in long-term or repeated interactions.	Qian and Wan (2025)
Human–AI co-creation	Vignette experiment	Social presence, commitment, reliance,	AI’s social presence enhances contribution willingness via team commitment and mutual interdependence.	Constrained to a text-based collaboration context, the broader need to belong was not examined.	Siemon et al. (2025)
Human–human co-creative	Survey and interview	Social support and belonging	The arts intervention improved connections, belonging, social ties and feelings of community.	Small sample and no control group limit conclusions; belonging-focused findings need further validation.	Henrickson et al. (2024)
Human–human co-creative	Large-scale analysis	Needs for achievement, power, and affiliation	Achievement and power needs suppressed climate effects, affiliation need mediated its impact on citizenship and participation.	Using cross-sectional data from one community, the study may face self-selection bias and omit key moderators.	Tan et al. (2024)

In co-creation contexts, individuals with a high need to belong are more likely to perceive human partners as providing emotional support and social interaction, thereby enhancing the perceived usefulness of the collaboration. Conversely, individuals with a low need to belong are more focused on task efficiency and knowledge advantages, making them more likely to perceive human–AI co-creation as more useful. In contrast, novelty is more dependent on AI’s cross-domain generation abilities and non-traditional thinking, rather than being influenced by the individual’s level of need to belong. Accordingly, this study proposes the following hypotheses:

*H3a*: The need to belong moderates the mediating effect of perceived usefulness. Specifically, for individuals with a low need to belong, human–AI co-creation enhances perceived usefulness more than human–human co-creation, thereby increasing co-creation intention; for individuals with a high need to belong, the difference in perceived usefulness between human–AI co-creation and human–human co-creation is not significant.

*H3b*: The need to belong does not moderate the mediating effect of perceived novelty. In other words, regardless of the level of the need to belong, human–AI co-creation always enhances perceived novelty more than human–human co-creation, thereby promoting co-creation intention.

To test these hypotheses, this study conducted Supplementary Study 4, aiming to further examine whether the need to belong moderates the mechanism through which co-creator type affects co-creation intention via perceived usefulness. Additionally, to eliminate the potential interference from the emotionally social tasks used in the first two studies, we chose cognitive-analytical tasks (Zhang et al., 2025) and included cognitive load as an alternative mediator to enhance the robustness and generalizability of the results.

### 6.1 Experimental design and participants

We adopted a mixed design with a between-subjects factor (co-creator type: human vs. AI) and a within-subjects factor (the need to belong). Via the Credamo platform, we recruited 350 participants with prior experience using generative AI; after attention checks, 294 valid responses remained. Among them, 53.1% were female; 75.1% were aged 21–40; and 81.3% held associate/bachelor’s degrees.

### 6.2 Procedure and measures

Participants were randomly assigned to the human–human co-creation or human–AI co-creation condition and read the following vignette: “You are participating in a creative task. Your goal is to write a popular-science article, including information gathering and writing. You will complete the entire creation process together with a human partner (vs. AI) to produce the final work.” The measurement procedure mirrored Study 2, with the additional assessment of the need to belong. The need to belong scale (Cronbach’s *α* = 0.743) was adapted from Leary et al. (2013). The full vignette and procedure are provided in Appendix C.

### 6.3 Results

We conducted mediation analysis using Hayes’s (2017) PROCESS macro (Model 4; bootstrapping = 5,000). The results were consistent with those of Studies 2 and 3: the total effect of co-creator type on co-creation intention was significant [*β* = 0.221, SE = 0.078, *t* = 2.84, *p* = 0.005, 95% CI (0.068, 0.375)], but the direct effect was not significant [*β* = −0.051, SE = 0.066, *t* = −0.77, *p* = 0.441, 95% CI (−0.180, 0.079)], indicating full mediation. Further analysis revealed that both novelty [*β* = 0.069, SE = 0.027, 95% CI (0.023, 0.130)] and usefulness [*β* = 0.220, SE = 0.050, 95% CI (0.127, 0.322)] played significant mediating roles, while cognitive load was not significant [*β* = −0.017, SE = 0.021, 95% CI (−0.049, 0.008)], ruling out the alternative explanation.

We conducted a mediated moderation analysis using PROCESS Model 7 (Hayes, 2017) with 5,000 bootstrap resamples. The results showed that the interaction between co-creator type and the need to belong did not significantly affect novelty [*β* = −0.193, SE = 0.150, *t* = −1.283, *p* = 0.201, 95% CI (−0.489, 0.103)], but had a significant negative effect on usefulness [*β* = −0.466, SE = 0.077, *t* = −6.024, *p* < 0.001, 95% CI (−0.618, −0.314)]. Conditional effect analysis revealed that at low levels of the need to belong, the positive effect of co-creator type on usefulness was strongest [*β* = 0.855, SE = 0.102, *t* = 8.427, *p* < 0.001, 95% CI (0.656, 1.055)], whereas at high levels of the need to belong, this effect was not significant [*β* = −0.008, SE = 0.101, *t* = −0.081, *p* = 0.936, 95% CI (−0.207, 0.191)].

**Figure 5:**
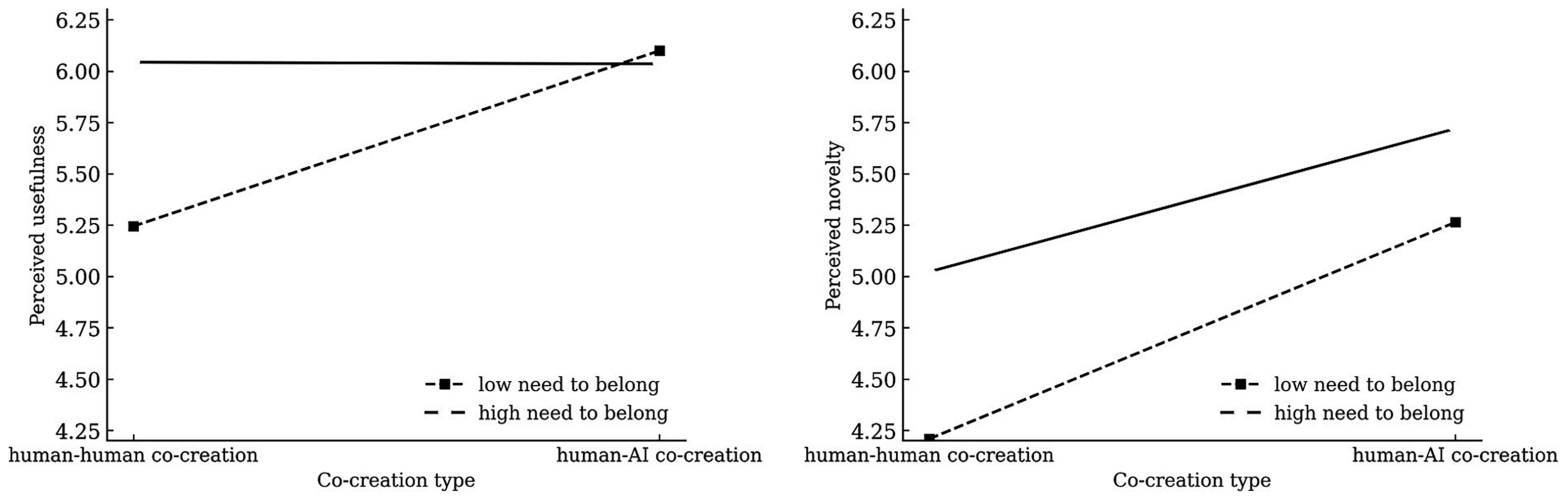
Interaction plot of the moderation effect.

Further moderated mediation analyses indicated that the indirect effect via perceived novelty was not moderated by the need to belong [*β* = −0.017, SE = 0.017, 95% CI (−0.058, 0.010)], whereas the indirect effect via perceived usefulness was significantly moderated [*β* = −0.264, SE = 0.057, 95% CI (−0.380, −0.158)]. Specifically, at low levels of the need to belong the indirect effect was significant (β = 0.484, SE = 0.086, 95% CI [0.326, 0.660]), whereas at high levels it was not [*β* = −0.005, SE = 0.057, 95% CI (−0.116, 0.109)]. *H3a* and *H3b* were supported (see Figure 5).

### 6.4 Discussion

Study 4 further corroborated the earlier findings in the context of cognitive analytical content. Moreover, it revealed a critical moderating role of the need to belong in the mediation via perceived usefulness. Specifically, the indirect effect of collaboration type on co-creation intention through perceived usefulness was stronger among individuals lower in need to belong and weaker among those higher in need to belong. These results delineate a social-motivational boundary for the usefulness pathway while maintaining the overall pattern observed in prior studies.

## 7 Conclusion and discussion

### 7.1 Research conclusions

This study systematically examined the effects of different co-creator types (human vs. AI) on co-creation intention and their underlying mechanisms through three scenario-based experiments (Studies 1, 2, and 4) and one semi-structured interview (Study 3). The specific conclusions are as follows.

First, relative to co-creating with humans, people are generally more inclined to engage in human–AI co-creation. This inclination primarily stems from AI’s marked advantages in enhancing perceived novelty and perceived usefulness. Situated within creativity theory (Runco and Jaeger, 2012), this pattern indicates that human–AI collaboration can raise novelty and usefulness concurrently rather than trading one off against the other, thereby strengthening intention. Human–AI co-creation not only feels more novel than traditional human–human co-creation but also exhibits distinctive practical value, thereby broadly increasing individuals’ co-creation intention. This conclusion holds across task types and study designs without reiterating study-specific details; in aggregate, whether in emotional social tasks (e.g., short-story creation and promotional-poster design) or in cognitive analytical content (e.g., popular-science writing), AI significantly elevates participants’ willingness to create through novel co-creation modes and efficient creative support. Moreover, the analyses rule out task self-efficacy and cognitive load as alternative mediators, reinforcing the centrality of perceived novelty and perceived usefulness within a creativity-theoretic account of co-creation intention.

Second, the perceived usefulness of co-creating with AI can be explained by three primary drivers: efficiency, value, and relationship. On efficiency, AI can generate a large number of ideas in a very short time; this high efficiency makes collaboration with AI especially attractive for complex, time-pressured tasks, where AI can deliver quick feedback and support. On value, drawing on a powerful knowledge base and diverse ways of thinking, AI helps break conventional creative frames and offers new perspectives and solution concepts—capabilities that are particularly salient in the creative industries. AI’s originality and cross-domain capacity not only provide fresh inspiration but also strengthen creators’ recognition of AI as a collaborative partner. Finally, along the relationship dimension, AI’s “de-interpersonalized” character affords a distinctive advantage: in human–AI co-creation, individuals need not worry about emotional volatility, communication barriers, or interpersonal conflict; this low emotional cost mode of collaboration smooths the creative process and increases participants’ satisfaction with AI as a partner.

Finally, the need to belong plays a critical moderating role in the mediation via perceived usefulness. The study shows that individuals low in the need to belong are more likely to perceive human–AI co-creation as more useful, which, in turn, increases their co-creation intention. In contrast, individuals high in the need to belong focus more on emotional support and social interaction, and therefore, their perceived usefulness increases to a lesser extent when co-creating with AI. This difference suggests that individuals’ social needs significantly moderate their preferences for different co-creator types: those low in the need to belong tend to prioritize task efficiency and the practicality of creative outcomes, while those high in the need to belong place greater value on emotional communication and social interaction with human partners. Thus, the need to belong not only plays an important role in the formation of co-creation intention but also offers a differentiated perspective on AI’s application in co-creation based on individual differences.

### 7.2 Theoretical contributions

This study is the first to systematically compare the effects of human and AI as co-creators on co-creation intention, filling a gap in previous research on preferences regarding human–AI collaboration. Existing studies have primarily focused on objective indicators such as creative efficiency, quality, and psychological effectiveness, with less attention given to individual preferences. For instance, Tang et al. (2024) examined the impact of human–AI co-creation on creative quality, and Huang et al. (2024) explored trust and acceptance in human–AI collaboration. However, these studies did not address the subjective question of “with whom are people more willing to co-create?” By directly comparing human and AI as co-creation partners, this study provides systematic empirical evidence, thus extending the understanding of co-creation preferences in the human–AI collaboration field.

This study further extends the application of creativity theory to the AI-collaboration context, distinctly differentiating itself from existing research that only explores the independent role of perceived novelty or perceived usefulness in isolation (Liu and Huang, 2025; Keith et al., 2024). It specifies perceived novelty and perceived usefulness as parallel mediating variables that form the cognitive pathway linking co-creator types to co-creation intention. Creativity theory posits that novelty and usefulness are core dimensions for evaluating creativity (Runco and Jaeger, 2012), providing the theoretical foundation for understanding outcomes in human–AI co-creation. Although prior work has touched upon perceptions of novelty and usefulness in AI collaboration, it has not integrated these two dimensions into a unified analytical framework nor revealed the internal mechanism through which they synergistically influence co-creation intention. The mediation model advanced here—where co-creator types influence co-creation intention through perceived novelty and perceived usefulness—rectifies the analytical limitation of “single perceptual dimension” in existing research, clarifies how human–AI co-creation enhances co-creation intention through a dual cognitive pathway rather than a single-dimensional effect, and thus deepens and expands the application boundary of creativity theory in the human–AI context.

The study also finds that AI, as a co-creation partner, not only increases participants’ perceived novelty of creative outputs but also significantly enhances their perceived usefulness. This result directly challenges the conventional assumption that “humans are more useful whereas AI is more novel” (Chen and Chan, 2024; Revilla et al., 2023), offering a new theoretical lens for adjusting role perceptions in human–AI collaboration. Traditional views hold that humans have the advantage in the practical usefulness of creative work, while AI mainly contributes innovative ideas. However, Doshi and Hauser (2024) show that generative AI elevates not only the novelty of ideas but also their usefulness. In line with this, our findings indicate that human–AI co-creation simultaneously boosts perceived novelty and perceived usefulness, breaking the cognitive bias of fragmented functions between the two and prompting a reassessment of human and AI roles in co-creation.

Finally, this study for the first time integrates social motivation (the need to belong) into the human–AI application framework of creativity theory, finding that the need to belong moderates the mediation via perceived usefulness, thereby revealing the crucial role of social motivation in the effects of AI co-creation. Baumeister and Leary (1995) argue that humans possess a strong need to belong, which shapes attitudes and behaviors in collaboration (Afota et al., 2024). Consistent with this view, Bergdahl et al. (2023) also find that individuals’ social motives significantly influence their acceptance of AI technologies and their willingness to collaborate. Our results show that when the need to belong is low, AI significantly increases the perceived usefulness of creative outputs, thereby enhancing co-creation intention; when the need to belong is high, this effect is not significant. These findings clarify the boundary conditions for the application of creativity theory in the human–AI collaboration context. By integrating a comprehensive framework where co-creator types operate through dual perceptual mediation, with social motivation acting as a moderator, they address the deficiency of existing research in exploring contextual boundaries and provide a more holistic theoretical reference for the development of theories in the field of human–AI collaboration.

### 7.3 Managerial implications

First, organizations introducing generative AI should clearly position it as an augmenting rather than replacing co-creation partner. Managers can help employees understand that AI’s primary function is to expand creative boundaries, enhance efficiency, and integrate diverse knowledge instead of substituting human thought. Creative platforms can incorporate human–AI co-creation progress bars or contribution visualization modules that highlight users’ creative input, thereby reinforcing their sense of agency and creative confidence. Interface cues, guidance texts, and feedback messages can emphasize human involvement and originality, shaping the perception of AI as a co-creator of inspiration rather than an automated author. These practical designs can reduce users’ resistance and foster positive engagement with AI systems.

Second, organizations promoting AI applications should account for employees’ psychological and motivational differences. Since individuals vary in their need for belonging, social interaction, and efficiency orientation, AI systems should provide differentiated and adaptive interface modes. For employees with high belonging needs, interfaces can include social interaction elements such as emotionally expressive AI dialogue, team-based co-creation spaces, or co-signature displays that convey joint authorship, helping maintain emotional connection and social presence. For those with low belonging needs, AI tools should emphasize functional simplicity, speed, and task efficiency through features like quick generation, personalized command memory, or modular output settings. Such adaptive design ensures that users’ motivational profiles align with system affordances, improving satisfaction and sustained co-creation engagement.

Finally, managers should proactively prevent the potential demotivating effects of AI by reinforcing human value and achievement. Creative teams can adopt phased co-creation mechanisms in which AI assists during the ideation and content generation stages, while human members retain responsibility for synthesis, judgment, and final innovation. Organizations should also establish recognition and reward systems that highlight human contributions—for instance, maintaining creator attributions in AI-assisted works, incorporating “human–AI collaboration quality” indicators into performance appraisals, and encouraging employees to share best practices in AI-assisted creativity. These actionable measures sustain intrinsic motivation, balance technological benefits with human needs, and help build a more human-centered and sustainable AI co-creation environment.

### 7.4 Limitations and future directions

First, Although our hypotheses were supported across varied experimental scenarios, these tasks still differ from fully authentic content co-creation. Future work will therefore move beyond survey vignettes to field experiments in organizational and platform settings, preregistered where feasible, to test behaviorally consequential outcomes and strengthen external validity. Building on the present triangulation, we will also broaden the qualitative inquiry to map additional candidate mechanisms that may shape partner preference, and then use targeted experimental manipulations to adjudicate among them in more complex tasks and diverse participant populations. This multi-method program will allow us to replicate the core effects under higher ecological realism, examine boundary conditions, and connect perceived usefulness to observed collaborative performance.

Second, this study focused on writing and image-generation tasks—domains where AI is widely used and professional barriers are relatively low—while paying less attention to more complex operations (e.g., model design, video production). The mechanisms underlying individuals’ co-creation intention may differ across task forms and warrant deeper investigation.

Third, external factors may drive dynamic changes in co-creation intention and merit further study. For example, as task complexity increases, individuals may prefer collaborators who excel at that task; thus, task complexity may influence individuals’ intention to engage in content co-creation with AI.

Fourth, this study did not conduct an exploratory analysis of the potential mechanisms underlying perceived usefulness. We focused on establishing the core effect and providing a targeted qualitative refinement of the construct rather than mapping a full process model. Future research will undertake deeper mechanism-focused work, beginning with broader qualitative exploration to surface candidate pathways and followed by targeted experimental tests that probe mediation and boundary conditions across more complex tasks and diverse samples.

Fifth, our samples were recruited in China via Credamo. This limits generalizability and raises cultural alternative explanations. Norms related to collectivism, power distance, and relational orientation may shape how people evaluate the usefulness of an AI partner and how willing they are to co-create. Future work should test whether the effects replicate across cultures with different value profiles. We recommend multi-country replications using matched procedures and measurement invariance checks, along with preregistration and harmonized materials. Field studies in workplaces and classrooms would increase ecological validity by observing partner choice and performance in real tasks. Studies that track behavior over time could examine longer term adoption and switching between co-creating with humans and co-creating with AI.

## Data Availability

The raw data supporting the conclusions of this article will be made available by the authors, without undue reservation.

## Footnotes

1
www.Credamo.com
