# Supplementary material for: From humans to AI: understanding why AI is perceived as the preferred co-creation partner
Source: Front Psychol. 2025 Dec 8;16:1695532. doi: 10.3389/fpsyg.2025.1695532 (PMC12722866; doi:10.3389/fpsyg.2025.1695532)
Supplement: Supplementary file 1 [file Supplementary_file_1.docx]

**Appendix A Study 1 Vignette and Procedure**

**1.****Vignette Text**

Imagine that you are about to complete a creative writing task with a partner. You need to collaboratively write a short story, including developing the plot and writing the text. You and your partner will use an online collaboration interface to brainstorm ideas, draft an outline, and polish sections of the story. The collaboration does not require real-time chat, but you can choose to adopt or ignore the partner’s suggestions as you see fit. The story genre can be contemporary or light science fiction. Please avoid gory or adult content. Ensure the plot is complete, characters are clear, and the ending is coherent. The recommended length is about 800–1,200 words, and the task should take around 15 minutes in total.

**Collaboration process:**

Stage 1: Generate a title and establish the main character(s) and their goal.

Stage 2: Design the plot structure.

Stage 3: Polish the language of a key scene and check for consistency.

If the partner provides multiple suggestions, you may choose the best option before continuing.

**2.Conditions**

**2.1 Human-Human Co-creation (Condition 1)**

You will collaborate with a human partner to complete the writing task. This partner is a writing enthusiast selected by the platform, with solid Chinese writing skills and basic narrative techniques. The platform will display the key points and suggestions provided by the partner at each stage of the task on the collaboration interface (including ideas for character setup, plot development, and language refinements). You may adopt, modify, or ignore the partner’s suggestions. The platform will automatically integrate both of your contributions into the final story. In subsequent questions, please answer with reference to this experience of co-creating with a human partner.

**2.2 Human-AI Co-creation (Condition 2)**

You will collaborate with an AI partner to complete the writing task. This partner is a general Chinese writing and editing system with reliable language generation abilities and basic narrative skills. The system will provide key points and suggestions at each stage in list form on the interface (including ideas for character setup, plot development, and language refinements). You may adopt, modify, or ignore these suggestions. The platform will automatically integrate your input and the AI’s input into the final story. In subsequent questions, please answer with reference to this experience of co-creating with an AI partner.

**3.Unified Task Instructions and Examples**

**Stage 1 Prompt:** Determine the protagonist and their desire, and specify one internal obstacle and one external obstacle for the protagonist. Example: The protagonist is a novice urban planner. Their desire is to revitalize the public spaces in an old town. An internal obstacle is the protagonist’s self-doubt. An external obstacle is a tight project deadline along with diverging opinions from local residents.

**Stage 2 Prompt:** Organize the story into a three-act structure. Example framework: Act I (Beginning): The protagonist receives the city renovation project and immediately faces opposition. Act II (Confrontation): The protagonist and the partner propose two opposite solutions and test their feasibility; an unexpected event forces them to integrate their ideas. Act III (Conclusion): In a community meeting, the protagonist finds a compromise solution, and through this process the protagonist experiences personal growth.

**Stage 3 Prompt:** Polish the language of a key scene in the story. Ensure the characters’ motivations are consistent from before to after, and that environmental details serve to advance the plot. Example sentence: “The rain-soaked cobblestones gleamed like thin silver sheets; residents’ voices intertwined at the mouth of the alley, and as the argument began the protagonist finally voiced their real concern.”

Note: The above examples are provided only to illustrate the task format and do not limit your own choices. Please base your decisions on the suggestions provided by your partner.

**4.Manipulation and Attention Checks**

**Comprehension Check (single-choice)**

“In this task, what type of partner were you collaborating with?”
A. Human partner;

B. AI partner;

C. I will complete the task by myself;

D. Not sure.

**Attention Check (single-choice)**

“Please read this carefully. This question is an attention check. To confirm you are paying attention, please select Option 2.”
A. Option 1;

B. Option 2;

C. Option 3;

D. Option 4.

If a participant answered the comprehension check or attention check incorrectly, the system prompted them to reread the scenario or terminated the survey.

**5. Scale Items**

All items below were rated on a **7-point Likert scale** (1 = completely disagree, 7 = completely agree).

**Partner Type Identification (manipulation check):**

“I just collaborated with a human partner.”

“I just collaborated with an AI partner.”
Note: Only the item corresponding to the actual condition was used for scoring; the other item served as a reverse-check for condition awareness.

**Perceived Novelty:**

“I feel that this co-creation mode is novel.”

“I feel that this co-creation mode is rare.”

“This co-creation mode is eye-opening to me.”

**Perceived Usefulness:**

“I feel that this co-creation mode is effective.”

“I feel that this co-creation mode is valuable.”

“I feel that this co-creation mode is appropriate.”

**Co-create Intention:**

“I am willing to collaborate with this partner.”

“I am satisfied with this co-creation mode.”

“I am willing to continue working with this partner in the future.”

**Process Experience and Control Variables:**

“I felt supported during the collaboration.”

“I had a high level of control over the final manuscript.”

“I was quite familiar with the writing task.”

**6. Demographics and Screening Information**

Participants were asked the following background and demographic questions at the end of the study:

**Generative AI usage experience:** “Please briefly describe your experience with generative AI.” (Response options: Never used, Occasionally used, or Frequently used. If “frequently used,” participants were asked to list the type of task for their most recent use. This question was used for sample segmentation and robustness analysis and did not affect compensation.)

**Gender:** (Male / Female / Other)

**Age:** (20 years or below; 21–25; 26–30; 31–35; 36–40; above 40)

**Education:** (High school or lower; Associate/Bachelor’s; Master’s; Doctorate)

**Occupation:** (Full-time student; Government or public institution employee; Corporate employee; Self-employed business owner; Freelancer)

Participants were informed that their personal information would be used only for academic research and sample segmentation analysis. All data would be processed anonymously and kept strictly confidential. Providing this information would not affect their compensation or future participation, and they could choose not to answer or withdraw at any time.

**7. Experimenter Notes**

The two condition texts were identical except for the wording of the partner type (human vs. AI).

The content was presented in the following order: Vignette Text, Condition Description, Unified Task Instructions, then Check Questions and Scale Items.

The comprehension check and attention check appeared immediately after the condition description text.

The manipulation check and all scale items were presented at the end of the survey.

Participants’ reading time for the scenario and response time for the questions were recorded for quality control.

**Appendix B Study 2 Vignette and Procedure**

**1.** **Vignette Text**

Imagine that you are about to complete a creative design task with a partner. You need to collaboratively create a promotional poster, which includes coming up with a slogan and designing the layout. You and your partner will use an online collaboration interface to brainstorm ideas, sketch a layout, and refine the copy. The collaboration does not require real-time conversation, but you can choose to adopt or ignore the partner’s suggestions as needed. The poster’s theme will be either a campus environmental campaign or a community public service event. The design should have clear information, well-coordinated elements, and a consistent style. A vertical single-page layout is recommended. The task should take about 12–15 minutes to complete.

**Collaboration process:**

Stage 1: Generate ideas and organize information – clarify the target audience, core message, and call-to-action.

Stage 2: Determine the layout framework and visual hierarchy – decide on the title, subtitle, main graphic elements, and use of whitespace.

Stage 3: Refine the slogan and details – polish wording, adjust alignments, and ensure images match the text.

If the partner provides multiple suggestions, you may choose the best option to proceed.

**2. Conditions**

**Human-Human Co-creation (Condition 1)**

You will collaborate with a human partner to complete the design. This partner is a design and copywriting enthusiast selected by the platform, with solid visual communication skills and basic layout abilities. The platform will display the key points and suggestions provided by the partner at each stage on the interface (including ideas for information organization, layout arrangement, and slogan wording). You may adopt, modify, or ignore the partner’s suggestions. The platform will automatically integrate both of your choices into the final poster design. Subsequent questions will refer to this experience of co-creating with a human partner.

**Human-AI Co-creation (Condition 2)**

You will collaborate with an AI partner to complete the design. This partner is a general Chinese visual-design and copy generation system with reliable key-point extraction and suggestion capabilities. The system will provide key points and suggestions at each stage in list form on the interface (including ideas for information organization, layout arrangement, and slogan wording). You may adopt, modify, or ignore these suggestions. The platform will automatically integrate your input and the AI’s input into the final poster. Subsequent questions will refer to this experience of co-creating with an AI partner.

**3. Unified Task Instructions and Examples**

**Stage 1 Prompt:** Clarify the target audience and the core message, and propose three candidate slogans.

Example key points: The target audience is current university students, and the core message is about waste sorting and exchanging items for rewards. Example slogans: “Today’s sorting makes tomorrow’s campus more beautiful.”; “Put the bottle in the right place, put environmental protection in your heart.”; “One small step in sorting, one giant leap for a greener campus.”

**Stage 2 Prompt:** Sketch the **layout framework** for the poster.

Example framework: Place the title at the top. The main visual (e.g., an illustration or a cluster of icons) is centered. A call-to-action button or event details (time and place) are placed at the bottom. Leave equal whitespace on the left and right sides to ensure readability.

**Stage 3 Prompt:** Refine the **slogan and details** of the poster.

Example guidelines: Start the title with a verb to increase its call-to-action force. In the subtitle, add details such as time and place. Ensure the image does not redundantly illustrate the text. Use no more than three primary colors in the design.

Note: The above examples are provided only to illustrate the task format and do not restrict your own creative choices.

**4.Manipulation and Attention Checks**

**Comprehension Check (single-choice):** “In this task, what type of partner are you working with?”

Options: A. Human partner; B. AI partner; C. I will complete the task alone; D. Not sure.

**Attention Check (single-choice):** “Please read carefully. This question is an attention check. To ensure you are paying attention, please select Option 2.”

**Options:** A. Option 1; B. Option 2; C. Option 3; D. Option 4.

If a participant answered the comprehension or attention check incorrectly, the system prompted them to reread the scenario or terminated the survey.

**5. Scale Items**

All items below were rated on a 7-point Likert scale (1 = completely disagree, 7 = completely agree).

**Partner Type Identification (manipulation check):**

“I just collaborated with a human partner.”

“I just collaborated with an AI partner.”
Note: Only the item matching the actual condition was kept for scoring; the other item served as a reverse-check.

**Perceived Novelty:**

“I feel that this co-creation mode is novel.”

“I feel that this co-creation mode is rare.”

“This co-creation mode is eye-opening to me.”

**Perceived Usefulness:**

“I feel that this co-creation mode is effective.”

“I feel that this co-creation mode is valuable.”

“I feel that this co-creation mode is appropriate.”

**Co-create Intention:**

“I am willing to collaborate with this partner.”

“I am satisfied with this co-creation mode.”

“I am willing to continue working with this partner in the future.”

**Task Self-Efficacy:**

“I am confident that I can complete this task.”

“I am confident that I can come up with good ideas.”

“I am confident that I can create a good design.”

**Process Experience and Control Variables:**

“I felt supported during the collaboration.”

“I had a high degree of control over the final design.”

“I was quite familiar with the design task.”

**6. Demographics and Screening Information**

The survey concluded with the following questions for participant background and demographics:

**Generative AI usage experience:** “Please briefly describe your experience with generative AI.” (Options: Never used, Occasionally used, Frequently used. If “frequently used,” participants listed the type of task they last used it for. This question was for post-hoc segmentation/analysis and did not affect compensation.)

**Gender:** (Male / Female / Other)

**Age:** (20 or below; 21–25; 26–30; 31–35; 36–40; above 40)

**Education:** (High school or below; Associate/Bachelor’s; Master’s; Doctorate)

**Occupation:** (Full-time student; Government/public sector; Corporate employee; Self-employed; Freelancer)

Participants were told that this information would only be used for academic research and sample segmentation. All personal data would remain anonymous and confidential. Providing this information would not influence their payment or future participation. Participants could skip any question or withdraw from the study at any time.

**7. Experimenter Notes**

The two version texts (human vs. AI condition) were identical except for the partner description (human or AI).

1. Presentation order of the materials was: Vignette and Procedure, Condition Description, Unified Task Instructions, followed by Check Questions and Scale Items.
2. The comprehension check and attention check were presented immediately after the condition description text.
3. The manipulation check and all scale items were presented at the end of the questionnaire.
4. Participants’ reading time for the scenario and answering time for the questions were recorded for quality control purposes.

**Appendix C Study 4 Vignette and Procedure**

**1.Vignette Text**

Imagine that you are about to complete a science popularization writing task with a partner. You need to work together on gathering content and writing, in order to produce a popular science article for the general public. You and your partner will use an online collaboration interface to conduct literature search and extract key points, build an outline and draft paragraphs, and perform language polishing and fact-checking. The collaboration does not require real-time discussion, but you can choose to adopt or ignore the partner’s suggestions as you see fit. The article’s topic will be a specific issue under one of three broad categories: health & everyday science, urban & environmental science, or digital technology & society. The writing should be accurate and easy to understand, logically clear, and it should avoid excessive jargon (any necessary technical terms should be explained). The recommended length is about 1,000–1,500 words, and the task should take approximately 15–20 minutes in total.

**Collaboration process:**

Stage 1: Sources and key points – identify the target audience and communication goal, gather 3–5 core scientific points and note relevant data or authoritative sources.

Stage 2: Outline and structure – construct a 3- or 4-paragraph structure, specifying an introductory hook, the scientific principles, examples of applications, and any cautions or limitations.

Stage 3: Draft and proofread – write two key paragraphs of the article, and unify terminology, check facts, and ensure proper citation formatting.

Throughout the process, the interface will display the partner’s list of key points and example sentences. At each stage, you will decide whether to adopt or discard the partner’s suggestions.

**2. Conditions**

**Human-Human Co-creation (Condition 1)**

You will collaborate with a human partner to complete the writing. This partner is a science-writing enthusiast selected by the platform, with solid Chinese writing skills and basic scientific literacy. The platform will display the key points and suggestions provided by the partner at each stage on the interface (including source hints, structural suggestions, and sentence-level polish recommendations). You may adopt, modify, or ignore the partner’s suggestions. The platform will automatically integrate both of your inputs into the final article. In subsequent responses, please refer to this experience of co-creating with a human partner.

**Human-AI Co-creation (Condition 2)**

You will collaborate with an AI partner to complete the writing. This partner is a general Chinese writing and editing system with reliable language generation capabilities and basic narrative skills. The system will provide key points and suggestions at each stage in list form on the interface (including source hints, structural suggestions, and sentence-level polish recommendations). You may adopt, modify, or ignore these suggestions. The platform will automatically integrate your input and the AI’s input into the final article. In subsequent responses, please refer to this experience of co-creating with an AI partner.

**3. Unified Task Instructions and Examples**

**Stage 1 Prompt:** Clarify the target audience and the communication goal, and list 3–5 core scientific points with their source types.

Example: The target audience is non-expert general readers, and the goal is to dispel common misconceptions and provide practical advice. Example core points: Circadian rhythm affects sleep quality and attention; regular exposure to natural light helps synchronize one’s internal clock; caffeine and blue light can delay sleep onset. Example source types: major review articles, official guidelines from authoritative organizations, and statistical reports.

**Stage 2 Prompt:** Construct the article structure.

Example framework: Start with a real-life scenario to introduce the topic. In the main body, use a simplified diagram or an analogy to explain the scientific mechanism, then provide two everyday application examples and discuss their limitations and risks. Conclude with a summary and actionable suggestions.

**Stage 3 Prompt:** Complete the writing of two key paragraphs and perform proofreading.

Example: Convert technical terms into layperson’s language and add a brief explanation (for instance, refer to the “internal biological clock” and explain how it relates to light exposure); when giving recommendations, include actionable steps and specify frequency ranges.

Note: The above examples are provided to illustrate the format of each stage and do not constrain your choices. Please make your decisions based on the partner’s suggestions.

**4. Manipulation and Attention Checks**

**Comprehension Check (single-choice):** “In this task, what type of partner are you collaborating with?”

Options: A. Human partner; B. AI partner; C. I will complete the task alone; D. Not sure.

**Attention Check (single-choice):** “Please read carefully. This question is an attention check. To ensure you are paying attention, please select Option 2.”

Options: A. Option 1; B. Option 2; C. Option 3; D. Option 4.

If the participant answered the comprehension or attention check incorrectly, the system prompted them to reread the scenario or ended the survey.

**5. Scale Items**

All items below were rated on a 7-point Likert scale (1 = completely disagree, 7 = completely agree).

**Partner Type Identification (manipulation check):**

- 1. “I just collaborated with a human partner.”
  2. “I just collaborated with an AI partner.”
     Note: Only the item corresponding to the actual condition was considered in scoring; the other was used as a reverse-identification check.

**Perceived Novelty:**

- 1. “I feel that this co-creation mode is novel.”
  2. “I feel that this co-creation mode is rare.”
  3. “This co-creation mode is eye-opening to me.”

1. **Perceived Usefulness:**
   1. “I feel that this co-creation mode is effective.”
   2. “I feel that this co-creation mode is valuable.”
   3. “I feel that this co-creation mode is appropriate.”
2. **Co-create Intention:**
   1. “I am willing to collaborate with this partner.”
   2. “I am satisfied with this co-creation mode.”
   3. “I am willing to continue working with this partner in the future.”
3. **Need for Belonging:**
   1. “In my work or life, I always hope to maintain close contact and communication with others.”
   2. “I enjoy working with others on tasks, and I believe team collaboration is more valuable than working alone.”
   3. “When completing a task, I feel more engaged and fulfilled if I have connection with other people.”
4. **Cognitive Load:**
   1. “While completing this task, I had to invest a lot of mental effort.”
   2. “I felt the pace was fast and time was pressing when working on the task.”
   3. “I put in a great deal of effort to accomplish the task well.”
5. **Process Experience and Control Variables:**
   1. “I felt supported during the collaboration.”
   2. “I had a high level of control over the final manuscript.”
   3. “I was quite familiar with the writing task.”

**6. Demographics and Screening Information**

At the end of the study, participants answered the following questions for screening and demographic purposes:

**Generative AI usage experience:** “Please briefly describe your experience using generative AI.” (Options: Never used, Occasionally used, Frequently used. If “frequently,” they listed the type of task last completed with AI. This question was used for sample stratification and robustness checks and did not affect the reward.)

**Gender:** (Male / Female / Other)

**Age:** (20 or below; 21–25; 26–30; 31–35; 36–40; above 40)

**Education:** (High school or below; College/University; Master’s; Doctorate)

**Occupation:** (Full-time student; Government or public sector; Corporate employee; Self-employed; Freelancer)

Participants were assured that this information would be used only for academic research and sample analysis. The data would remain anonymous and confidential. Providing or withholding personal information would not affect their compensation or subsequent participation. They were free to skip any question or exit the study at any time.

**7. Experimenter Notes**

1. Aside from the partner type wording, the texts for the two conditions were identical.
2. The scenario content was presented in the following order: Vignette and Procedure and Objectives, Condition Description, Unified Task Instructions, then Check Questions and Scale Items.
3. The comprehension check and attention check questions appeared immediately after the condition description text.
4. The manipulation check and all scale items were placed at the end of the questionnaire.
5. Participants’ scenario reading time and response time were recorded for quality control.
